# Preparation of Chitooligosaccharides with Specific Sequence Arrangement and Their Effect on Inducing Salt Resistance in Wheat Seedlings

**DOI:** 10.3390/polym17091194

**Published:** 2025-04-27

**Authors:** Jingwen Li, Anbang Li, Yupeng Li, Siqi Zhu, Lin Song, Song Liu, Ronge Xing, Kecheng Li

**Affiliations:** 1 College of Biological Engineering, Qingdao University of Science and Technology, Qingdao 266042, China; lijingwen223@163.com; 2 CAS and Shandong Province Key Laboratory of Experimental Marine Biology, Institute of Oceanology, Chinese Academy of Sciences, Qingdao 266071, China; lianbang23@mails.ucas.ac.cn (A.L.); yupengli2023@163.com (Y.L.); 18255914694@163.com (S.Z.); sliu@qdio.ac.cn (S.L.); xingronge@qdio.ac.cn (R.X.); 3 Laboratory for Marine Drugs and Bioproducts, Qingdao Marine Science and Technology Center, Qingdao 266237, China

**Keywords:** chitooligosaccharides, sequence arrangement, wheat, plant salt-resistance induction

## Abstract

Chitooligosaccharides (COS) exhibits good activity of inducing plant resistance, but the structure–activity relationship is still unclear. In this study, chitin oligosaccharides (CHOS) with a degree of polymerization (DP) of 2~6 were used as raw materials. Three deacetylases (NodB, *Vc*COD, and *Ar*CE4A) were employed to prepare three different sequence-arranged COSs, namely N-COS, C-COS, and A-COS, and their structures were characterized by infrared spectroscopy, high-performance liquid chromatography, and mass spectrometry. Further studies were conducted on inducing the plant salt resistance of the three different sequence-arranged COSs on wheat seedlings. The results showed a sequence-dependent effect of COS inducing plant salt resistance. Among them, A-COS exhibited the best activity. When sprayed at a concentration of 10 mg/L on wheat seedlings under salt stress for 3 days, the leaf length of the wheat seedlings sprayed with A-COS was recovered, and the wet mass and dry mass were recovered by 20.40% and 6.64%, respectively. Following the enhancement of proline accumulation, the malondialdehyde content decreased by 34.75%, and the Na^+^/K^+^ ratio also exhibited a significant reduction, thereby alleviating salt stress-induced damage. This study was the first to demonstrate the effect of COS with specific sequences on inducing plant salt resistance, providing a theoretical basis for the development of a new generation of efficient COS plant biostimulator.

## 1. Introduction

Salt stress is a common abiotic stress and is becoming an important factor affecting global food security [1,2]. Salt stress induces ionic toxicity, which disrupts the uptake of essential elements by plants, while the concomitant osmotic imbalance caused by high salinity leads to reduced stomatal conductance and a sharp decline in photosynthetic efficiency. At the same time, the oxidative stress induced by salt stress will produce a large amount of reactive oxygen species (ROS), causing lipid peroxidation and protein damage, ultimately leading to plant growth arrest or even death [3].

Current strategies to mitigate salt stress include exogenous chemical application, soil microenvironment optimization, agricultural water conservation technologies, and the cultivation of salt-tolerant cultivars [4]. Among these, exogenous chemical application is of particular importance due to its operational efficiency, targeted precision, and biocompatibility, working through direct interaction with stressed plants to activate antioxidant defense systems and enhance endogenous salt tolerance mechanisms. For example, the foliar application of salicylic acid (SA) effectively mitigates salt stress-induced malondialdehyde (MDA) accumulation in sunflower seedlings by activating the antioxidant defense system to scavenge ROS and attenuate lipid peroxidation-mediated cellular damage [5]. In addition, some natural polysaccharides also have good resistance-inducing effects. For example, the exogenous application of trehalose not only reduced MDA accumulation but also enhanced K^+^ uptake capacity in salt-stressed tomato seedlings, ultimately improving their growth performance [6]. This indicates that the exogenous application of some natural polysaccharides is an effective treatment method to counteract the negative effects of salt stress.

Chitin, a biomass-derived natural polysaccharide, composed of β(1→4)-glycosidic linked N-acetylglucosamine units, is the major component of the cell walls of various fungi and the exoskeletons of crustaceans and insects, serving as a critical biomolecule that triggers pathogen-associated molecular pattern (PAMP) responses through specific recognition by plant immune systems [7]. COS is the degradation product of deacetylated chitin and possesses good solubility, biocompatibility, and various biological activities such as antibacterial [8], the induction of chromium resistance [9], and salt tolerance [10]. COS has been reported to enhance salt tolerance in soybean seedlings through proline accumulation and lipid peroxidation mitigation under saline conditions, while simultaneously increasing shoot fresh biomass and promoting nutrient reserve deposition in plant tissues [11]. The induction of salt resistance in plants by COS has attracted extensive research attention, and it is a very promising plant biostimulator.

The molecular structure of COS determines its biological activity. COS is composed of alternating N-acetylglucosamine (**A**) and glucosamine (**D**), and the degree of acetylation (DA) is an important parameter affecting the biological activity of COS. Li et al. prepared COSs of three different DAs (0%, 32.2%, and 100%), and found that the fully deacetylated COS better promoted plant growth activity, which could promote the elongation of wheat roots and increase the stomatal conductance and increase the net photosynthetic rate and the biomass accumulation of wheat [12]. Using microwave-assisted KOH degradation, Fu et al. prepared COS with deacetylation degrees of 63.79%, 72.12%, 79.34%, and 88.15%, respectively. The results of the germination experiments on soaked wheat seeds showed that COS with a deacetylation degree of 72.12% could be the most effective in promoting the germination of wheat seeds, which was mainly achieved through increasing the activity of β-amylase and protease [13]. However, most COSs of different DAs were prepared by chemical methods in previous studies. The random distribution of monomers **A** and **D** in COS chains leads to the instability of the reported activity, and the structure–activity relationship between different acetyl sites and the biological activity of COS is not clear.

In recent years, chitin deacetylase has evoked increasing interest and could precisely remove one or more acetyl groups, thereby preparing COS with defined non-random acetylation sequences [14,15]. Zhu et al. prepared COSs using the deacetylase ScCDA2 from *Saccharomyces cerevisiae*, which selectively deacetylates the acetyl group at the non-terminal position of the sugar chain while retaining the acetyl group at the reducing end. The products were characterized by HPLC-ESI-MS and found to be seven COSs (**DAAA**, **ADAA**, **AADA**, **DDAA**, **DADA**, **ADDA**, and **DDDA**), thus providing an enzymatic strategy for the synthesis of specific COSs [16]. Hao et al. synthesized three chitobiose sequences (**AD**, **DA**, and **DD**) from an **AA** substrate using two deacetylases (NodB and *Vc*COD) and demonstrated that the different sequence architectures differentially modulate the antioxidative activity through the systematic evaluation of hydroxyl radical, superoxide radical, and DPPH scavenging activities coupled with reducing power assays [17]. Basa et al. also used these two deacetylases (NodB and *Vc*COD) in combination with chitinase and chitosanase to prepare several chitotetraoses with different sequences, **ADDD**, **DADD**, **DDDA**, and **DDDD**. It was found that **ADDD** and **DADD** showed strong stimulating activity on the oxidative burst of rice cells [18].

In summary, differences in the sequence arrangements of COSs lead to variations in their biological activity. However, the effect of different sequence arrangements of COS on its ability to induce salt resistance in plants remains unclear. In this study, a homogeneous sequence of chitin oligosaccharide (CHOS) was used as raw material, and three deacetylases with different deacetylation modes (NodB, *Vc*COD, and *Ar*CE4A) were employed to perform enzyme-catalyzed deacetylation. Three COSs with different sequence arrangements (N-COS, C-COS, and A-COS) were obtained. After the foliar application of low-concentration COS to wheat seedlings under salt stress, the physiological index, osmotic regulators content, MDA content, antioxidant enzyme activity, and Na^+^/K^+^ content were determined. The sequence effect on COS-induced wheat salt resistance was revealed, which provides crucial support for deciphering the sequence–activity relationship of COS in plant resistance induction. The clarification of the sequence arrangement of COS and its ability to induce salt resistance will help the development of the COS series of efficient antagonists, and the combination of COS with modern agricultural technologies such as nano-sized objects will help it play a better role in the field of agricultural production [19].

## 2. Materials and Methods

### 2.1. Materials

Chitin oligosaccharides with a degree of polymerization (DP) of 1~6 were purchased from the Tokyo Chemical Industry Co., Ltd. (Tokyo, Japan). The NodB sequence (NCBI acc. No. AJW76244.1) and *Vc*COD sequence (NCBI acc. No. AAF94439.1) were sourced from the NCBI database and provided to Sangon Biotech (Shanghai, China) Co., Ltd. for gene optimization and synthesis. And *Ar*CE4A was improved according to the method of Tuveng et al. [20], and its sequence was provided to GenScript Biotech Co. (Nanjing, China) for gene optimization and synthesis. *E. coli* BL21 (DE3) competent cells were purchased from Sangon Biotech (Shanghai, China) Co., Ltd. and used to express chitin deacetylases. A Malondialdehyde (MDA) Content Assay Kit, Peroxidase (POD) Activity Assay Kit, and Catalase (CAT) Activity Assay Kit were bought from Solarbio (Beijing, China). A Superoxide Dismutase (SOD) assay kit (WST-1 method) was bought from Nanjing Jiancheng Bioengineering Institute (Nanjing, China). All other chemicals and reagents were of analytical grade.

### 2.2. Preparation of COSs with Chitin Deacetylases

#### 2.2.1. Heterologous Expression and Purification of Chitin Deacetylases

*E. coli* BL21 (DE3) was transformed into NodB, *Vc*COD, or *Ar*CE4A plasmids, respectively, and then grown in LB medium containing 1 μg/mL of ampicillin sodium (Amp) at 37 °C. When the OD600 reached 0.6~0.8, 0.1 mM isopropyl-β-D-thiogalactopyranoside (IPTG) was added to the culture medium for induction, and *E. coli* was cultured overnight at 16 °C. The cells were disrupted by sonication in Banding Buffer (50 mM Tris, 150 mM NaCl, pH = 8.0) and centrifuged at 4 °C to remove cell debris. Then, the protein was collected by gradient elution with 20~250 mM imidazole eluent in a Ni-agarose gel column. The collected eluent was analyzed by 12% SDS-PAGE, and the protein concentration was determined by the Coomassie brilliant blue dye method [21].

#### 2.2.2. Preparation of COSs with Specific Sequence Arrangement

The monosaccharide in commercial chitin oligosaccharides was removed by alcohol precipitation to obtain CHOS with DPs of 2~6. NodB reacted with CHOS at a mass ratio of 1:20 overnight in a buffer (20 mM Mops, 10 mM DTT, 1 mM MnSO_4_, pH = 7.5), and the product was purified by dialysis to obtain COS with the first deacetylation at the non-reducing-end residue (N-COS). *Vc*COD reacted with CHOS at a mass ratio of 1:20 overnight in a buffer (10 mM NH_4_HCO_3_, pH = 7.5), and the product was purified by dialysis to obtain COS with the deacetylation at the second residue from the non-reducing end (C-COS). The reaction of *Ar*CE4A with CHOS was carried out according to the method recorded by Tuveng et al. [21], and the reaction time was 48 h. After the reaction, the product was purified by dialysis to obtain multi-site deacetylated COS (A-COS).

### 2.3. Characterization

Powdered solid samples were detected in the range of 4000–500 cm^−1^ using an FTIR spectrometer, Thermo Scientific Nicolet iS10 (Thermo Fisher Scientific, Waltham, MA, USA), in ATR mode.

The LC-2030C 3D Plus HPLC system (SHIMADZU, Kyoto, Japan), equipped with the evaporative light detector (Essentia ELSD-16), was used for the analysis of COS by hydrophilic interaction liquid chromatography (HILIC). The chromatographic column was SHIMADZU ShimNex HE Amide (5 μm, 4.6 mm × 150 mm). The mobile phase was a binary mobile phase consisting of 20% ammonium formate solution and 80% acetonitrile. The flow rate was 1 mL/min, and the column temperature was 25 °C. The sample was filtered through a 0.22 nm needle filter prior to analysis.

ESI-MS was performed using LTQ Orbitrap XL (Thermo Fisher Scientific, Waltham, MA, USA). The ESI source voltage was 4 kV, the sheath gas was 10 arbs, the scan gas was 0 arbs, and the capillary temperature was 275 °C.

### 2.4. Plant Materials and Treatment

Wheat seeds (Jimai 22) were germinated in the dark at 25 °C for 24 h and then transferred to a culture cup. The temperature was maintained at 25/20 °C, the day/night cycle was 14/10 h, and the humidity was maintained at 65~70%. The Hoagland nutrient solution was replaced every other day. When the second leaf was fully developed, the wheat seedlings were randomly divided into seven experimental groups. Each experimental group had three parallels, including a control group (CK, no NaCl was applied, but distilled water was sprayed with 0.01% Tween-20), a negative control group (NaCl, 100 mM NaCl was applied, and distilled water was sprayed with 0.01% Tween-20), and a positive control group (SA, 100 mM NaCl was applied, and 100 mg/L of salicylic acid was sprayed with 0.01% Tween-20). The concentration of NaCl was in reference to Kashif et al. [22]. And the four sample groups were the CHOS group, N-COS group, C-COS group, and A-COS group. Each parallel group had 30 wheat seedlings. In total, 100 mM salt stress was applied to the culture solutions of the four groups of wheat, and the corresponding samples were sprayed at a concentration of 10 mg/L (0.01% of Tween-20 was added to the solution). Each parallel group was sprayed with 30 mL of sample solution each time, and the spraying process lasted for three days. After 10 days of treatment, the corresponding physiological and biochemical indexes of wheat seedlings were determined.

### 2.5. Determination of the Growth Parameters

After 10 days of treatment, the leaf length, root length, and fresh mass of fresh wheat seedlings were measured, and the dry mass was measured by baking wheat seedlings at 105 °C to a constant weight.

### 2.6. Determination of the Proline Content

After spraying the leaves with drugs for 10 days, wheat leaves were taken at the same position in the mortar, and 3% sulfosalicylic acid solution was added to the ice bath to extract proline. The free proline content was determined by the ninhydrin method [23], and the absorbance value of the microplate reader at 520 nm was recorded. The standard curve was drawn with L-proline as the standard to quantify the concentration.

### 2.7. Determination of the MDA Content

MDA is a product of lipid peroxidation in plants. The MDA content is used to determine the level of lipid peroxidation in plants. The same parts of wheat leaves were frozen and ground in liquid nitrogen, and the MDA content was determined using the MDA Content Kit.

### 2.8. Determination of Antioxidant Enzyme Activities

After 10 days of treatment, the same parts of wheat leaves were frozen in liquid nitrogen and homogenized to extract crude enzymes. The activities of T-SOD, POD, and CAT in wheat seedlings were determined by the T-SOD Activity Assay Kit, POD Activity Assay Kit, and CAT Activity Assay Kit, respectively.

### 2.9. Determination of Na^+^ and K^+^ Contents

After 10 days of treatment, the same parts of the wheat leaves were frozen in liquid nitrogen and ground. The contents of Na^+^ and K^+^ in wheat leaves were determined by Thermo ICPOES7200 (Thermo Fisher Scientific, Waltham, MA, USA). The emission power was 1.15 kW, the carrier gas was argon, the ion gas flow rate was 15 L/min, the auxiliary gas flow rate was 15 L/min, the nebulizer flow rate was 0.75 L/min, the detection mode was axial observation, and the calibration type was linear.

### 2.10. Statistical Analysis

All experimental data were analyzed by ANOVA using SPSS software (version 27.0), and the significant difference was detected by Duncan multiple range test (*p* < 0.05). Each dataset is expressed as the mean ± standard deviation of three independent replicates. Different letters (a, b, c...) on the same parameter indicate significant differences at the *p* < 0.05 level.

## 3. Results

### 3.1. Heterologous Expression and Purification of Deacetylase

**Figure 1:**
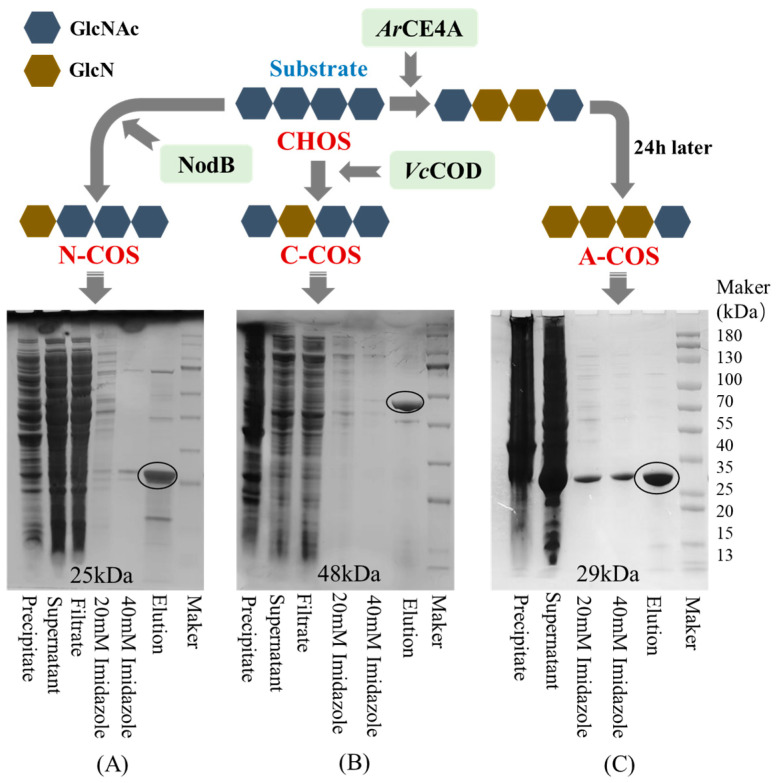
The results of the heterologous expression and purification of deacetylase and the deacetylation action sites (taking chitotetraose as an example). (**A**) SDS-PAGE of NodB; (**B**) SDS-PAGE of *Vc*COD; (**C**) SDS-PAGE of *Ar*CE4A.

To prepare accurate and non-random sequence COS, the deacetylases selected in this study for single-site deacetylation are NodB from *Sinorhizobium meliloti* and *Vc*COD from *Vibrio cholerae*. NodB removes the acetyl group at the first residue from the non-reducing end, while *Vc*COD removes the acetyl group at the second residue from the non-reducing end [24,25]. Since NodB and *Vc*COD deacetylate the acetyl groups on the non-reducing end, to clarify the specific effect of acetylation sites on the activity of COS, this study also selected a multi-site deacetylase (*Ar*CE4A) from *Marine Arthrobacter*. *Ar*CE4A preferentially removes the acetyl groups on the internal sugar chain, then removes the acetyl group at the first residue from the non-reducing end, and finally retains the acetyl group at the reducing end. As shown in Figure 1, NodB, *Vc*COD, and *Ar*CE4A are successfully expressed in *E. coli*. After purification using Ni-sepharose, SDS-PAGE analysis shows that the apparent molecular weights of NodB, *Vc*COD, and *Ar*CE4A are close to the expected 25 kDa, 48 kDa, and 29 kDa, respectively.

### 3.2. Characterization of COSs with Specific Sequence Arrangements

**Figure 2:**
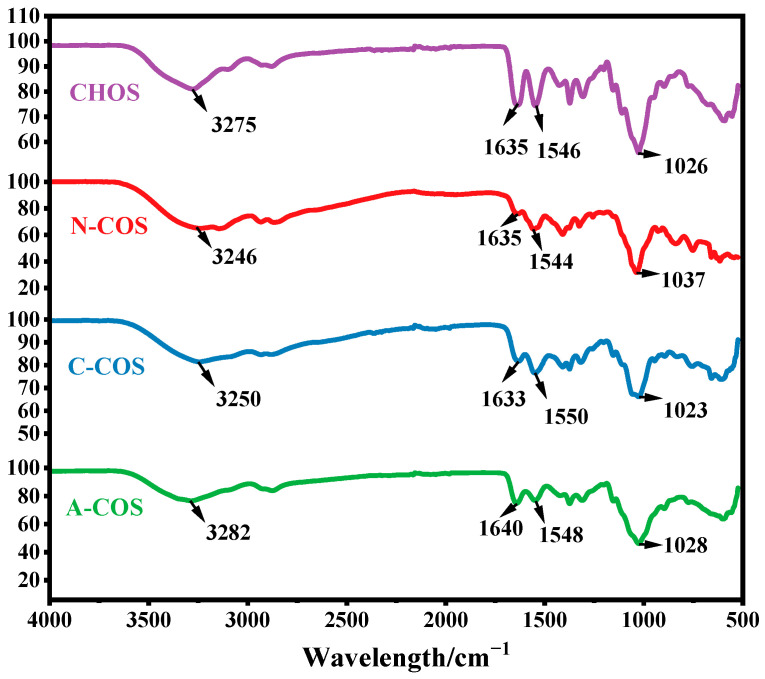
The FTIR spectra of CHOS, N-COS, C-COS, and A-COS.

The FTIR spectra of CHOS, N-COS, C-COS, and A-COS were analyzed, as shown in Figure 2. The characteristic peaks at 3275, 1635, 1546, and 1026 cm^−1^ correspond to the stretching vibration of −OH, the stretching vibration of C=O, the bending vibration of N-H, and the vibration of sugar ring in CHOS, respectively. CHOS exposes the amine groups after removing the acetyl groups, and the signal peak at 3200–3400 cm^−1^ changes from sharp to smooth due to the overlap of the stretching vibration of −OH and −NH_2_. N-COS, C-COS, and A-COS showed this change after deacetylation, proving that the acetyl groups were successfully removed. However, due to the three types of COSs only removing a small amount of acetyl groups, the basic structure is still similar to the substrate CHOS, and the bending vibration peak at 1597 cm^−1^ corresponding to the free amine group in the full deacetylated COS infrared spectrum is not observed [26].

**Figure 3:**
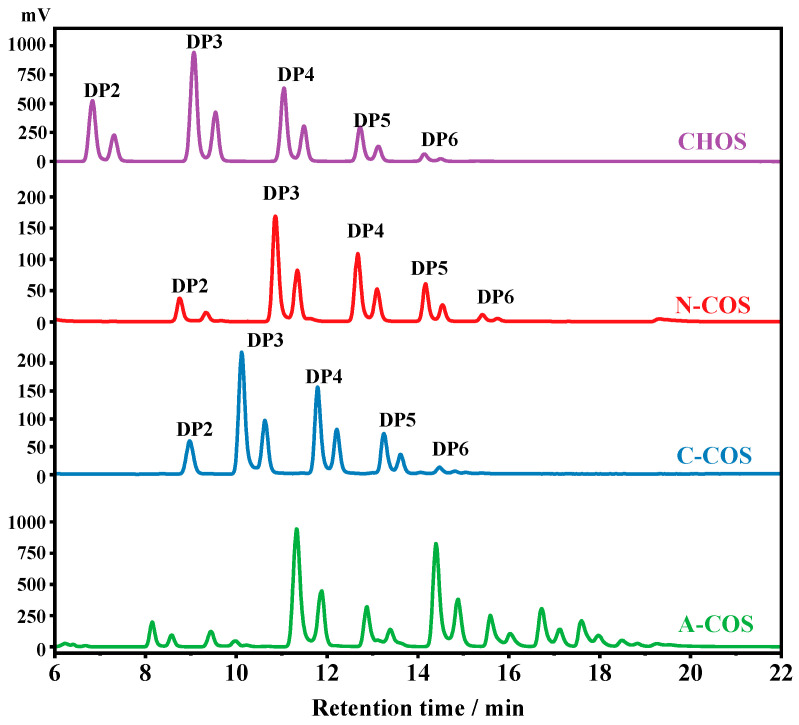
The HPLC spectra of CHOS, N-COS, C-COS, and A-COS.

The HPLC analyses of CHOS, N-COS, C-COS, and A-COS were further performed, as shown in Figure 3. Since the HPLC uses a hydrophilic interaction system, the retention time of the components with lower molecular weight or smaller degrees of polymerization (DP) in CHOS is short. Therefore, the oligomers in CHOS are attributed to be chitin oligosaccharides with DP2, DP3, DP4, DP5, and DP6, respectively. After deacetylation, the amine groups exposed by deacetylase have better hydrophilicity than the acetylamino groups, and the affinity with the hydrophilic column is stronger, so the retention time is delayed. The retention time of N-COS and C-COS as mono-deacetylated COS is significantly delayed compared to CHOS, indicating that the two deacetylases have good activity and no substrate residue. The retention times of DPs of 2~6 after NodB and *Vc*COD deacetylation are also different, indicating that the two produced COSs are isomers. It also shows that the NodB and *Vc*COD expressed in this study have very good specificity and can specifically remove acetyl groups from the first and second residues at the non-reducing end, respectively. As a multi-site deacetylated COS, the retention delay of A-COS is more obvious. Compared with the removal of one acetyl group from N-COS and C-COS, the retention time of all components is less than 16 min, while A-COS still has components after 16 min, which also proves that the enzymatic reaction of *Ar*CE4A removes more acetyl groups from CHOS.

**Figure 4:**
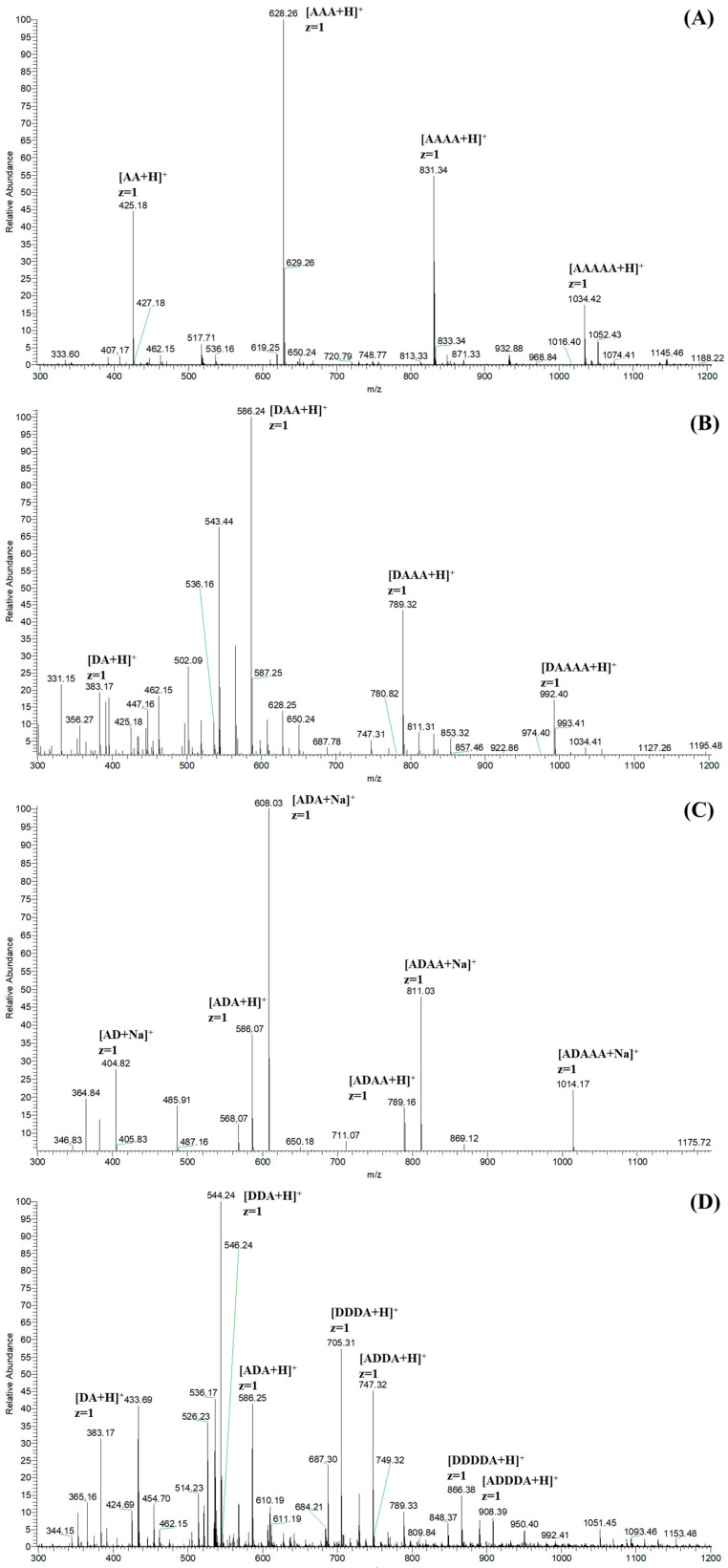
The ESI-MS spectra of CHOS (**A**), N-COS (**B**), C-COS (**C**), and A-COS (**D**).

The ESI-MS spectra of CHOS, N-COS, C-COS, and A-COS were carried out for the exact composition analysis. As shown in Figure 4A, the ion peaks of the main components are labeled as [M + H]^+^ ion peaks corresponding to **AA**, **AAA**, **AAAA**, and **AAAAA**, respectively. Taking the ion peak with a *m*/*z* of 425.18 as an example [27], the calculation method is 425 = 2 × 221(GlcNAc) − 18(H_2_O) + 1(H). Figure 4B shows the main components of N-COS (*m*/*z* 383), with one acetyl group removed, which are attributed to **DA**, **DAA**, **DAAA**, and **DAAAA**. N-COS and C-COS are isomers, both being mono-deacetylated COSs, so they show the same *m*/*z* signals in the ESI-MS spectra (Figure 4C). According to the deacetylation site of the *Vc*COD, C-COS mainly contains **AD**, **ADA**, **ADAA**, and **ADAAA**. The ion peaks of **DA**, **D_2_A**, **DA_2_**, **D_3_A**, **D_2_A_2_**, **D_4_A**, and **AD_3_A** are shown in Figure 4D. Based on the characteristics of *Ar*CE4A, the chitin deacetylase preferentially removes internal acetyl groups but has no activity on reducing terminal residues. Therefore, the products in A-COS are identified as **DA**, **DDA**, **ADA**, **DDDA**, **ADDA**, **DDDDA**, and **ADDDA**.

### 3.3. Wheat Growth Index

**Figure 5:**
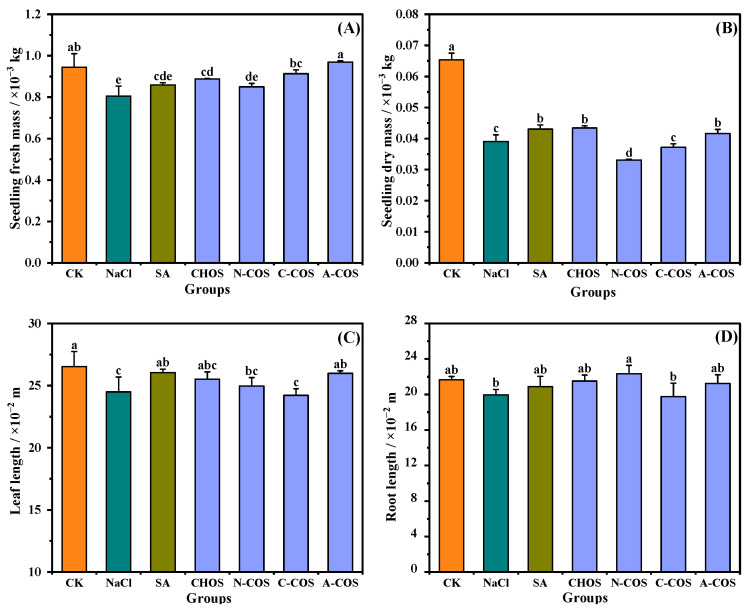
Effects of different treatment groups on growth indexes. (**A**) Seedling fresh mass; (**B**) seedling dry mass; (**C**) leaf length; (**D**) root length. Different letters (a,b,c,d,e) indicate significant differences at *p* < 0.05 on the same parameter.

As shown in Figure 5, 100 mM salt stress significantly reduced both fresh mass and dry mass in wheat seedlings. The foliar application of CHOS (*p* = 0.007, <0.05), C-COS (*p* = 0.001, <0.05), and A-COS (*p* < 0.001) significantly enhances fresh mass accumulation in wheat seedlings, with A-COS exhibiting the most pronounced efficacy by restoring fresh mass to the CK group. Salt stress exerts more pronounced impacts on dry mass, reducing dry mass to 59.73% of control levels, while CHOS (*p* = 0.002, <0.05) and A-COS (*p* = 0.045, <0.050) treatments significantly ameliorate this suppression compared with the NaCl group. Notably, the dry mass restorations of CHOS and A-COS formulations achieve comparable activity to the positive control group (salicylic acid, SA). Changes in the leaf length and root length of wheat seedlings under salt stress are not obvious. In summary, all growth indexes of wheat seedlings sprayed with A-COS under salt stress can be maintained at good levels.

### 3.4. Proline Content

**Figure 6:**
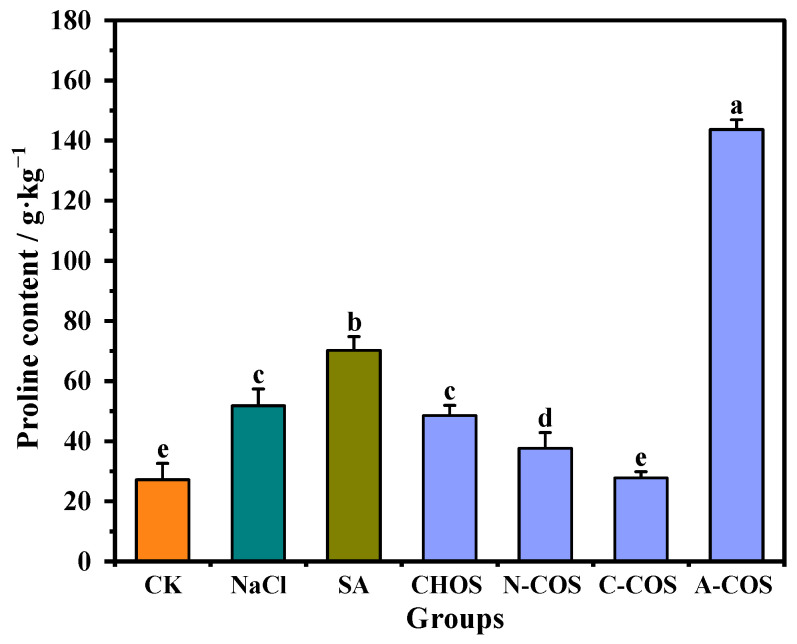
Effect of different treatment groups on proline content. Different letters (a,b,c,d,e) indicate significant differences at *p* < 0.05 on the same parameter.

Proline emerges as a critical multifunctional osmolyte in plant salt stress responses, serving a dual role as an indicator of ionic stress and a protective solute that stabilizes protein biosynthesis and metabolic processes. And it effectively prevents protein aggregation, the level of which is a key phenotypic indicator of superior salt tolerance in wheat varieties [28]. As shown in Figure 6, salt stress triggers a 90.34% increase in proline accumulation compared to control conditions, demonstrating the critical role of this compatible solute in the osmotic adjustment of wheat seedlings under saline conditions. After the SA application, the proline content of wheat seedlings is further increased, and the proline content of wheat seedlings in the SA group is 2.58 times higher than that in the CK group. A large gap appears in the changes in proline content after COS spraying, in which A-COS has an extremely significant promoting effect on the accumulation of proline in wheat seedlings, rising to 5.28 times that of the CK group.

### 3.5. MDA Content

**Figure 7:**
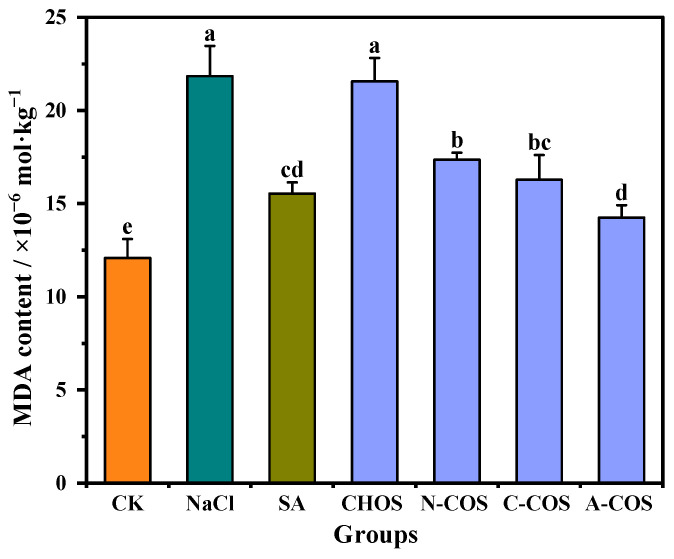
Effects of different treatment groups on MDA content. Different letters (a,b,c,d,e) indicate significant differences at *p* < 0.05 on the same parameter.

Salt stress induces ROS overaccumulation in plants, which subsequently attack membrane-bound unsaturated fatty acids, thereby initiating lipid peroxidation cascades that generate cytotoxic metabolites including MDA and compromise membrane integrity. This oxidative assault disrupts cellular homeostasis by impairing developmental processes and metabolic functions, with MDA serving as a critical biomarker of lipid peroxidation. Reducing the accumulation of MDA in the plant can effectively alleviate oxidative damage in the plant [29]. The MDA contents of wheat seedlings in different treatment groups are shown in Figure 7. Salt stress results in increased lipid peroxidation in wheat seedlings, and the MDA content in the salt stress group is elevated by 80.64% compared with that in the CK group. The exogenous spraying of SA significantly alleviates this condition, and the MDA content is only 28.51% higher than that of the CK group. The foliar application of distinct COSs differentially modulates salt stress-induced oxidative damage in wheat seedlings. The treatment of CHOS maintains MDA levels comparable to the salt-stressed group, indicating a limited capacity to mitigate lipid peroxidation. N-COS, C-COS, and A-COS applications significantly reduce MDA levels by 20.51%, 25.47%, and 34.75%, respectively. Notably, A-COS demonstrates SA-equivalent efficacy in ameliorating lipid peroxidation, establishing it as a potent antioxidant intervention against salt-induced membrane damage.

### 3.6. Antioxidant Enzyme Activities

**Figure 8:**
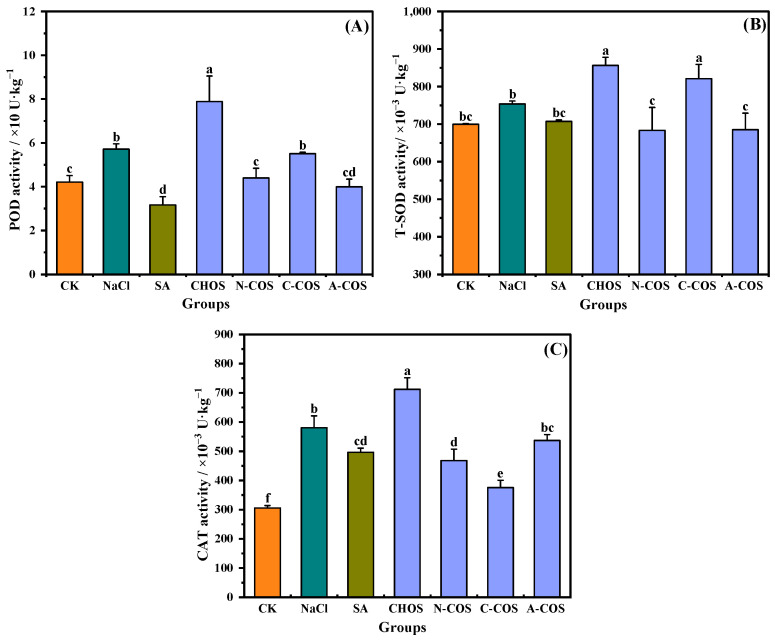
Effects of different treatment groups on antioxidant enzyme activities. (**A**) POD activity; (**B**) T-SOD activity; (**C**) CAT activity. Different letters (a,b,c,d,e,f) indicate significant differences at *p* < 0.05 on the same parameter.

ROS produced by plants under salt stress are scavenged by plant antioxidant enzymes, which are important components of oxidative defense in plants. POD, SOD, and CAT, as the main antioxidant enzymes, effectively reflect the stress response of the oxidative defense system to salt stress in plants [30]. SOD converts superoxide to hydrogen peroxide, CAT and POD function by scavenging hydrogen peroxide, and an effective dynamic equilibrium will be maintained between the activities of antioxidant enzymes and ROS in plant cells. As shown in Figure 8, POD (*p* = 0.004, <0.05) and CAT (*p* < 0.001) are activated under salt stress, showing an increase in enzyme activities. The foliar application of SA causes the activities of POD (*p* < 0.001) and CAT (*p* = 0.004, <0.05) to exhibit different degrees of regression. For wheat seedlings in the foliar-sprayed sample group, distinct samples show different effects. POD (*p* < 0.001), SOD (*p* = 0.002, <0.05), and CAT (*p* < 0.001) show an increase in activity in the presence of CHOS, while in the presence of A-COS, they all show a regression in activity, and the magnitude of this regression is consistent with that of SA.

### 3.7. Na^+^ Content and K^+^ Content

**Figure 9:**
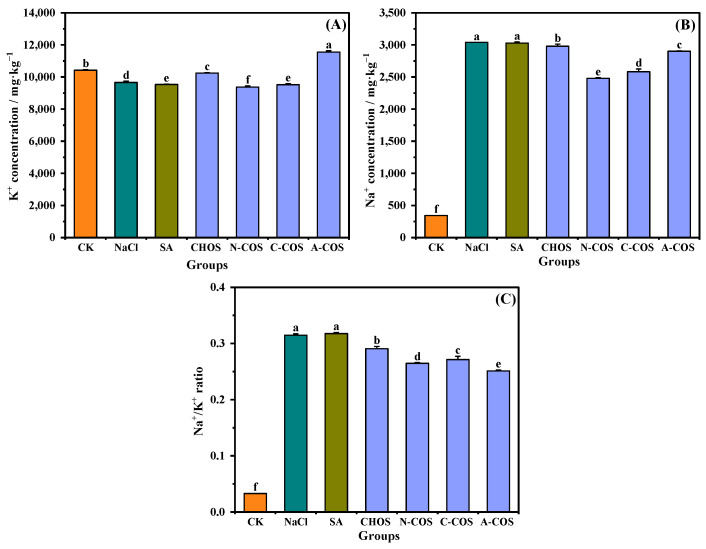
Effects of different treatment groups on Na^+^ and K^+^ contents. (**A**) K^+^ concentration; (**B**) Na^+^ concentration; (**C**) Na^+^/K^+^ ratio. Different letters (a,b,c,d,e,f) indicate significant differences at *p* < 0.05 on the same parameter.

Salt stress-induced Na^+^ hyperaccumulation in plant cells disrupts ionic homeostasis through competitive uptake via non-selective K^+^ channels and high-affinity transporter proteins in root systems. This Na^+^/K^+^ antagonism critically impairs the uptake of K^+^, which is an essential regulator for maintaining cellular osmotic equilibrium and sustaining photosynthetic machinery functionality [31]. As shown in Figure 9, salt stress induces a significant imbalance in the ionic homeostasis of wheat seedlings, a marked Na^+^/K^+^ ratio (*p* < 0.001), through enhanced Na^+^ influx and suppressed K^+^ assimilation. The foliar application of CHOS and COS significantly attenuates this ratio, with the hierarchy of efficacy being A-COS > N-COS > C-COS > CHOS. Notably, A-COS exhibits superior K^+^ uptake enhancement, thereby mitigating Na^+^-induced cytotoxicity and restoring membrane potential stability.

## 4. Discussion

Salt stress impacts plant growth via diverse physiological mechanisms. The application of A-COS significantly improves multiple physiological parameters in wheat seedlings, completely restoring shoot fresh weight to normal levels. Wheat seedlings exhibit high sensitivity to salt stress, especially during the early stages of growth. Under salt stress, plants initiate metabolic regulation such as organic matter conversion for osmoregulation and ion homeostasis. However, these compensatory mechanisms are insufficient to counteract the severe damage to photosynthetic assimilation caused by salt stress. Consequently, carbon allocation efficiency is significantly reduced, leading to a significant decrease in dry weight accumulation, as documented in previous studies [32,33]. Although the A-COS treatment failed to fully restore dry weight to the level of the unstressed control, it significantly increased dry matter accumulation to values comparable to those of the positive group treated by SA. This is consistent with the available evidence that COS can mitigate salt stress by regulating redox balance and nutrient uptake efficiency, thus supporting the maintenance of biomass under unfavorable conditions [34]. The beneficial effects may be attributed to the COS-mediated mitigation of salt-induced osmotic stress and membrane electrolyte leakage, coupled with improved cellular expansion and photosynthetic efficiency, which contributes to the increase in the fresh mass and dry mass of the plant [35,36].

The application of COS can effectively alleviate the damage caused by salt stress to plants. In this study, three distinct COSs exhibited varying efficacy in stress alleviation. Among them, the proline content in the wheat seedlings of the A-COS group increased several times, accompanied by a significant reduction in MDA levels. Previous studies have shown that COS triggers the expression of proline biosynthesis genes (i.e., OsP5CS, OsP5CR, Osδ-OAT) and inhibits the expression of proline-degrading enzyme OsProDH [37]. We also observed an increase in proline in wheat seedlings in the A-COS group, which is supposed to be a result of upregulation of proline synthesis-related genes. Functioning as both osmolyte and antioxidant, the accumulated proline directly participates in ROS scavenging, thereby mitigating oxidative damage and reducing cellular MDA accumulation [38]. Secondly, COS treatment was found to enhance cytoplasmic free Ca^2+^ levels and activate MAPK cascades, subsequently upregulating genes associated with phytohormone signal transduction [39,40]. This hormonal signaling network activates comprehensive antioxidant defense mechanisms, effectively controlling ROS levels and attenuating membrane lipid peroxidation. In this study, it is hypothesized that the decrease in antioxidant enzyme activity in wheat seedlings after spraying the COS is possibly caused by the reduction in ROS generation, resulting in a decrease in the stress demand of antioxidant enzymes (SOD, POD, and CAT), rather than the impairment of their functions. This possibility is rooted in previous studies reporting that the activity of antioxidant enzymes in wheat seedlings is balanced with the degree of lipid peroxidation [30,41]. When ROS in plant cells are effectively controlled, the activity of antioxidant enzymes will return to the normal level, which is similar to the situation where COS derivatives of γ-aminobutyric acid ameliorate salt stress in wheat [42]. N-COS and C-COS belong to a single deacetylated COS, and structurally, they are more similar to fully acetylated CHOS. Therefore, they have a weaker stimulating effect on proline-related metabolic enzymes.

Salt stress leads to an increase in Na^+^ absorption and a decrease in K^+^ absorption in plants. All three COSs effectively ameliorate cellular Na^+^/K^+^ homeostasis in wheat seedlings, with A-COS demonstrating superior activity. Research indicates that COS enhances the expression of SOS1 and NHX2 genes in plants, both of which are critical for sodium ion regulation. SOS1 mediates cellular Na^+^ efflux to the extracellular space, while NHX2 compartmentalizes intracellular Na^+^ into vacuolar compartments. This dual mechanism not only mitigates sodium toxicity but also synergistically enhances K^+^ uptake, collectively improving plant ionic homeostasis under saline stress conditions [43]. Notably, A-COS treatment in this case markedly enhanced K^+^ assimilation and ameliorated cellular Na^+^/K^+^ homeostasis. The improvements mediated by COS may result from upregulation of the expression of SOS1 and NHX2 genes, encoding critical sodium compartmentalization transporters involved in the vacuoles and thereby mitigating sodium toxicity, which is consistent with findings reported in prior research [44]. The pronounced efficacy of A-COS also correlates specifically with a significant accumulation of the osmoregulatory metabolite proline in treated seedlings. This compatible solute facilitates ionic balance through the dual mechanisms of membrane stabilization and reactive oxygen species scavenging [45,46].

By analyzing the various indicators of wheat seedlings, it is found that A-COS had a more excellent induction of salt stress resistance than N-COS and C-COS, which is attributed to the change in the arrangement pattern of the COS sequence. When the acetyl groups within the COS sugar chain are deacetylated, it causes a change in the overall hydrophobicity of the sugar chain, thereby affecting the interaction between COS and the binding proteins on the plant cell surface [47]. However, the acetyl groups at the reducing ends of these three COSs in this case remain unchanged. Therefore, the relationship between the reducing-end residue of COSs and their ability to induce salt resistance activity in plants requires further investigation.

## 5. Conclusions

In this study, three COSs with different sequence arrangements, namely N-COS, C-COS, and A-COS, were successfully produced by chitin deacetylases NodB, *Vc*COD, and *Ar*CE4A. Their roles in alleviating salt stress in wheat seedlings were systematically investigated. The results revealed that all three COSs mitigated salt-induced damage to varying extents, but their mechanisms of action differed significantly. The multi-site deacetylated COS (A-COS) exhibited the most pronounced salt stress alleviating activity. Specifically, A-COS was used to reduce ROS in wheat seedlings by promoting the accumulation of proline and attenuating lipid peroxidation and oxidative stress. Concurrently, it promoted K⁺ uptake, thereby alleviating osmotic imbalance and ion toxicity. A-COS effectively attenuated the accumulation of reactive oxygen species (ROS) in wheat seedlings through two mechanisms: (1) the enhancement of proline biosynthesis to counteract oxidative stress; (2) the suppression of lipid peroxidation cascades. At the same time, this treatment improved K⁺ intake, thereby restoring ionic homeostasis by mitigating Na^+^-induced osmotic imbalance and cellular toxicity. In contrast, single-site deacetylated COSs (N-COS and C-COS) did not promote proline accumulation in plants while reducing lipid peroxidation damage. Therefore, the removal of internal acetyl groups in COS chains is supposed to be critical for the induction of salt tolerance in plants, clearly establishing the positional specificity of deacetylation sites as a determinant of COS bioactivity. Unfortunately, we did not prepare COSs with deacetylation at the reducing end. COS-induced salt resistance in plants is an extremely complex process, and the exact mechanism needs to be further investigated. The effect of A-COS on plants under long-term salt exposure also needs to be conducted in order to reveal the ecological relevance of the COS application in agriculture.

## Data Availability

Data are contained within the article.

## Abbreviations

The following abbreviations are used in this manuscript:

COS	Chitooligosaccharides
ROS	Reactive oxygen species
SA	Salicylic acid
MDA	Malondialdehyde
PAMP	Pathogen-associated molecular pattern
**A**	N-acetylglucosamine
**D**	Glucosamine
DA	Degree of acetylation
CHOS	Chitin oligosaccharide
POD	Peroxidase
CAT	Catalase
SOD	Superoxide Dismutase
